# Optical-Thermally Excited Graphene Resonant Mass Detection: A Molecular Dynamics Analysis

**DOI:** 10.3390/nano11081924

**Published:** 2021-07-26

**Authors:** Xing Xiao, Shang-Chun Fan, Cheng Li, Yu-Jian Liu

**Affiliations:** 1 School of Instrumentation and Optoelectronic Engineering, Beihang University, Beijing 100191, China; liuyujian@buaa.edu.cn; 2 Key Laboratory of Quantum Sensing Technology, Ministry of Industry and Information Technology, Beijing 100191, China; 3 Shenzhen Institute of Beihang University, Shenzhen 518063, China

**Keywords:** graphene resonator, mass detection, molecular dynamics, thermal actuation

## Abstract

In consideration of the presented optical-thermally excited resonant mass detection scheme, molecular dynamics calculations are performed to investigate the thermal actuation and resonant mass sensing mechanism. The simulation results indicate that an extremely high temperature exists in a 6% central area of the graphene sheet exposed to the exciting laser. Therefore, constraining the laser driving power and enlarging the laser spot radius are essential to weaken the overheating in the middle of the graphene sheet, thus avoiding being burned through. Moreover, molecular dynamics calculations demonstrate a mass sensitivity of 214 kHz/zg for the graphene resonator with a pre-stress of 1 GPa. However, the adsorbed mass would degrade the resonant quality factor from 236 to 193. In comparison, the sensitivity and quality factor could rise by 1.3 and 4 times, respectively, for the graphene sheet with a pre-stress of 5 GPa, thus revealing the availability of enlarging pre-stress for better mass sensing performance.

## 1. Introduction

Graphene-based nanoelectromechanical systems (NEMS) offer rich prospects for delicate sensing applications, including accelerometers [1], biomedical detection [2], and gas measurement [3]. In particular, the high surface-area-to-volume ratio, ultra-high Young’s modulus [4], and excellent electronic characteristics [5] give graphene-based NEMS devices unprecedented potential for mass sensing. After Geim and co-workers fabricated monolayer graphene by mechanical exfoliation [6], they verified the applicability of graphene in gas molecule detection [3], which relies on the change of local carrier concentration in graphene. Inspired by this work, many researchers have dedicted themselves to this kind of research. While achieving a limit of detection as low as parts per billion (ppb) [7,8], the constraints of this method, such as the susceptibility to temperature and the inapplicability to neutral macromolecules, have also been addressed. Meanwhile, the development of graphene resonators [9,10,11,12] has motivated scholars to adopt an alternative method using the mechanical frequency shift of graphene induced by the adsorbates to realize accurate minute mass measurement.

Before the advent of graphene resonators, SiC and carbon nanotubes were used as resonators for mass sensing, with the resolutions of zeptogram (10^−21^ g) and yoctogram (10^−24^ g) mass, respectively [13,14,15,16,17]. In contrast, the two-dimensional structure endows graphene with greater potential for subtle mass sensing. For example, Chen et al. [12,18] measured the shifts in graphene resonant frequency with the evaporation and deposition of pentacene on the devices, and their results showed that both the adsorbed mass itself and the tension induced by the mass played important roles in the frequency shift. Recently, Muruganathan et al. [19,20] presented zeptogram-level mass sensing for Hydrogen and Argon mixed gas using a top-gated graphene resonator. They found that the frequencies of graphene resonators decreased from 95 MHz to 90 MHz with the injection of a Hydrogen and Argon gas mixture, and the quality factor decreased from 45 to 15. These results indicated the possibility of graphene nanomechanical resonators for lightweight mass detection. Meanwhile, the theoretical outcomes about graphene resonant mass detectors also flourished. For example, Duan et al. [21] adopted a hybrid structure of graphene sheets supported by carbon nanotubes as a resonator and then performed molecular dynamics (MD) simulations to demonstrate an ultrahigh mass resolution up to 1 yoctogram. Then Han et al. [22] used MD calculation to investigate the possibility of mass measurement based on the edge mode of graphene resonators, and the results showed that the mass sensitivity corresponding to the edge mode was about three times higher than that of the fundamental mode. Additionally, other approaches using nonlinear vibration [23], introducing vacancies [24], and adjusting capacitive force [25] have been attempted to improve the performance of graphene-based resonant mass sensors. However, most of these investigations were focused on the relationship between resonant frequencies and adsorbed mass, rather than providing a systematic detection scheme.

Herein, a scheme of graphene resonant mass sensing based on laser thermal actuation and Fabry-Perot interference measurement is presented. A molecular dynamics investigation is then performed to study the thermal actuation and resonant mass detection mechanism. The resonant mass detector model is scaled down to one-thousandth in the MD calculation to make the time consumption acceptable, and the number of atoms in the MD model was above 5 × 10^5^, much more than in our previous studies [26,27]. The temperature distribution of the graphene sheet exposed to the actuation laser is calculated, and then the central overheating in the graphene sheet is avoided by constraining the laser power and enlarging the heating spot radius. Moreover, water molecules are introduced as the adsorbed mass. The frequencies of graphene sheets at different relative humidities, ranging from 0 to 100% at 20 °C are calculated by MD, and the mass sensitivity is demonstrated to be 214 kHz/zg. Furthermore, the degradation of the resonant quality factor caused by the adsorbed water molecules is observed, and the stronger pre-stress is proved to contribute to the improvement of both the quality factor and the sensitivity.

## 2. Models and Methods

### 2.1. Schemes of the Optical-Thermally Excited Graphene Resonant Mass Detector

**Figure 1:**
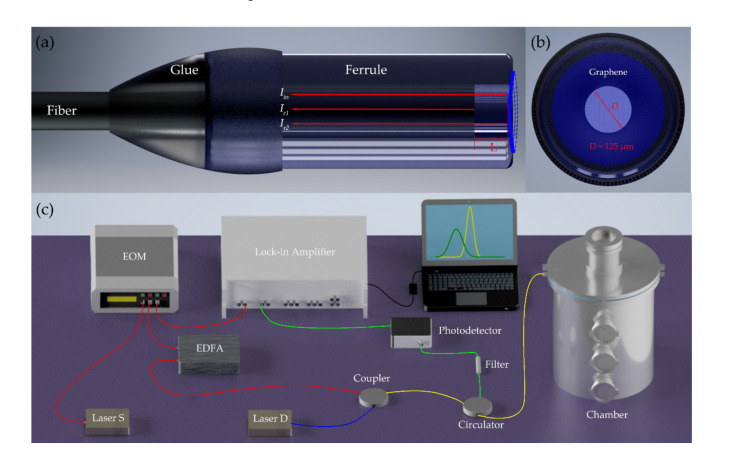
(**a**) Front view and (**b**) lateral view of the schematic diagram of the F-P resonant probe. (**c**) Scheme of the optical-thermal actuation setup for resonance measurement.

The presented resonant mass sensor was based on the adsorbates induced frequency shifts of graphene resonators. Figure 1a,b show the front and lateral view of the graphene resonator, respectively, which was a piece of monolayer graphene suspended on the end face of a zirconia ferrule, and the diameter of this tubular hole through the ferrule was 125 μm. The laser-thermal method was adopted to actuate the oscillation of the suspended graphene sheet, and its vibration was detected through the Fabry-Perot interference method. The whole beam path is depicted in Figure 1c. A distributed feedback (DFB) laser S with a wavelength of 1550.12 nm, which provided the thermal excitation, was modulated by an electro-optic modulator (EOM), and the swept modulation signal was given by a lock-in amplifier (HL2FI). The following erbium-doped fiber amplifier (EDFA) was used to compensate for the amplitude attenuation of laser S. The DFB laser D with a wavelength of 1551.72 nm was used to detect the vibration of the membrane. The actuation and detection lasers were coupled through a 2 × 1 coupler, and then delivered to the F-P cavity of the resonant graphene probe, which is shown in Figure 1a. The two beams of the reflected laser from the end face of fiber (*I_r_*_1_) and the surface of graphene membrane suspended on the ferrule (*I_r_*_2_), respectively, were sent to the filter through the circulator. The actuation laser was filtered, while the detection laser was captured by a photodetector (PD) with a bandwidth of 200 MHz, and the converted electrical signal was processed by the lock-in amplifier and shown on screen.

**Figure 2:**
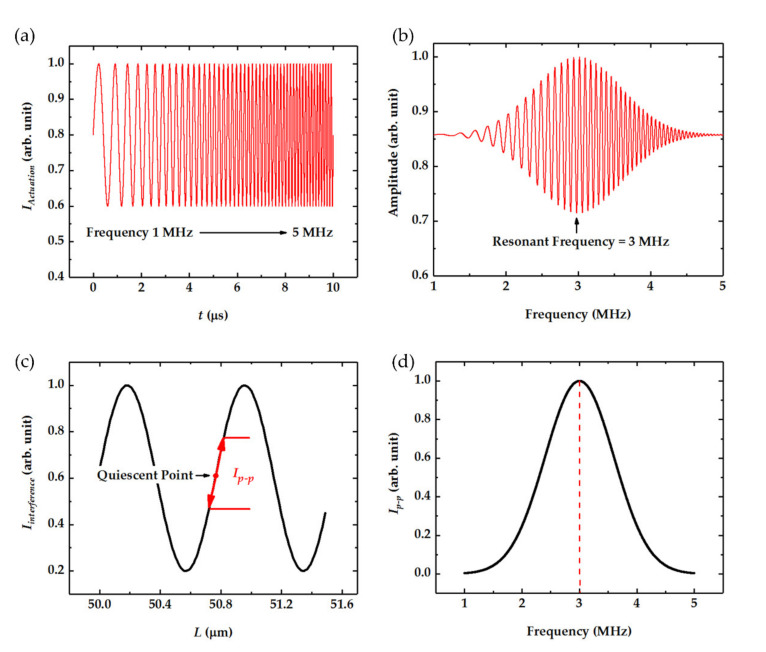
The signal flow of the graphene vibration actuation and detection. (**a**) The intensity of the actuation laser modulated by a signal swept from 1 MHz to 5 MHz. (**b**) The vibration amplitude of graphene under swept excitation. (**c**) The interferometer intensity versus the F-P cavity length. (**d**) The peak-to-peak interferometer intensity versus the actuation frequency.

The intensity of the actuation laser was modulated by a signal swept from 1 MHz to 5 MHz, as shown in Figure 2a. When exposed to this periodically modulated laser, the suspended graphene sheet would start vibrating in the same beat, and the vibration amplitude peaked when modulation frequency reached its eigenfrequency, as can be seen from Figure 2b. On the other hand, the vibration of graphene would change the F-P cavity length *L*, thus changing the interferometer intensity of the two reflected beams of *I_r_*_1_ and *I_r_*_2_, as noted in Figure 1a. The relationship of the interferometer intensity and the F-P cavity length is presented in Figure 2c. Furthermore, if the quiescent point was chosen properly as shown in Figure 2c, the peak-to-peak value of interferometer intensity would be linearly related to the vibration amplitude. Finally, the relationship between the peak-to-peak intensity and the actuation frequency could be obtained as shown in Figure 2d, which presents the amplitude–frequency response of the graphene sheet.

### 2.2. Models and Process of Molecular Dynamics Simulations

Molecular dynamics simulations [28,29,30] have been widely used to investigate the mechanism of thermal actuated circular graphene resonators. In MD simulations, the velocities and positions of every atom are figured out, and the evolution of the whole system is then obtained. To bring the calculation consumption within reach, the size of the simulation model was scaled to one-thousandth in this study.

**Figure 3:**
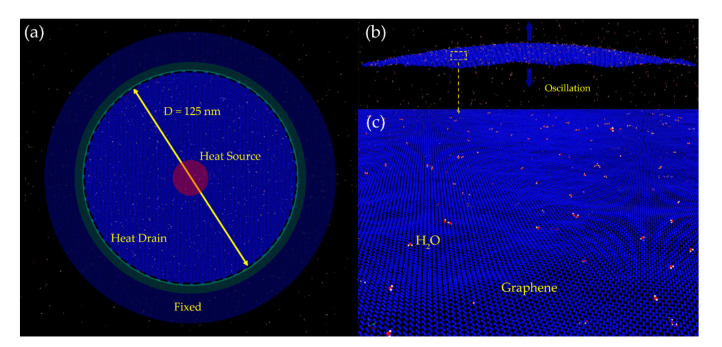
(**a**) MD model of the peripherally clamped circular graphene resonator for mass sensing. (**b**) An overview of the mechanical fundamental eigenmode of the peripherally clamped circular graphene. (**c**) The features of the graphene surface with water molecules serving as adsorbed mass.

The modeling schematic diagram of a peripherally clamped circular graphene sheet is shown in Figure 3. The diameter of the suspended part of the graphene sheet was 125 nm (one-thousandth of the truth), and the number of atoms in this system reached 5 × 10^5^, which far exceeds most relevant MD simulations that have been reported [31,32,33,34,35]. The interactions between these carbon atoms were described by the adaptive intermolecular reactive empirical bond order (AIREBO) [36,37,38], and the optimized Brenner potential parameter set [39] was considered in order to achieve a better fit to the thermal characteristics of graphene. The MD calculation was performed with the help of the Large-scale Atomic/Molecular Massively Parallel Simulator (LAMMPS) package from Sandia National Laboratories (Albuquerque, NM, USA). Before every section of MD simulation, the system structure was optimized through an equilibration process with a time step of 1 fs under the NPT ensemble, where the number of atoms, the pressure (1 atm), and the temperature (293 K) were kept constant. This investigation focused on the thermal distribution of graphene exposed to the laser, and the frequency shifts of graphene with water molecules adsorbed on its surface, as set out below.

#### 2.2.1. Thermal Distribution

We used a non-equilibrium MD (NEMD) calculation to study the thermal effect of graphene when exposed to an actuation laser. The laser spot was located at the center of the graphene sheets, which served as the heat source, as shown in Figure 3a. Atom velocities in this area were scaled at every step according to

(1) $$v_{i,t+1}=v_{i,t}{\left(1+\frac{\Delta Q}{E_{i,t}}\right)}^{1/2}$$

where Δ*Q* is the amount of heat added per atom; *E_i_* is kinetic energy for atom *i*; *v_i,t_* is the velocity of atom *i* at time *t*, and *v_i,t_*_+1_ is the corresponding velocity at the next step. Correspondingly, the atoms in the heat drain area had updated velocities, as follows

(2) $$v_{i,t+1}=v_{i,t}{\left(1-\frac{\Delta Q}{E_{i,t}}\right)}^{1/2}$$

After the heat flow from the center to boundary had attained a steady state, the velocities of every atom were recorded, and the temperature distribution was induced from the atom velocities averaged over time and space according to

(3) $$\frac{3}{2}k_{B}t=\frac{1}{2}mv_{rms}^{2}$$

where *t* is the temperature; *k_B_* is the Boltzmann constant; *m* is the atom mass, and *v_rms_* is the square root of the mean square of the atom velocities.

#### 2.2.2. Mechanical Frequencies

To actuate the oscillation of the graphene sheet in MD calculation, a cosinoidal initial velocity profile along the radius was employed, which can be described as

(4) $$v_{z}=v_{0}\cos\left(\frac{\pi r}{D}\right)$$

where *r* is the distance to the middle; *D* is the diameter of graphene, and *v*_0_ is set to 1 Å/ps, which is small enough to ensure linear flexural oscillation [27]. When the graphene started to vibrate, the movements of every atom were traced, and the overall vibration mode is shown in Figure 3b. After that, Fourier transform was employed to determine the resonant frequency of the graphene. To investigate the effect of adsorbed mass on the resonant frequency, water molecules were chosen as adsorbates and introduced to the MD simulation system, as depicted in Figure 3c, and the interaction between graphene and water molecules was described using the Lennard–Jones potential [40] expressed as

(5) $$E_{L-J}=4\varepsilon \left[{\left(\frac{\sigma }{r}\right)}^{12}-{\left(\frac{\sigma }{r}\right)}^{6}\right]$$

The first-principles study revealed that the adsorption energy between graphene and H_2_O was 0.047 eV [41,42,43], which was used as the potential well *ε* in Equation (5), and the zero-crossing distance *σ* for the potential was set to 3.16 Å. Under this potential, water molecules were attached to the surface of graphene as depicted in Figure 3c. After that, the adsorbate-induced resonant frequency shift could be calculated for further analysis.

## 3. Results and Discussion

### 3.1. Thermal Distribution under Laser Actuation

**Figure 4:**
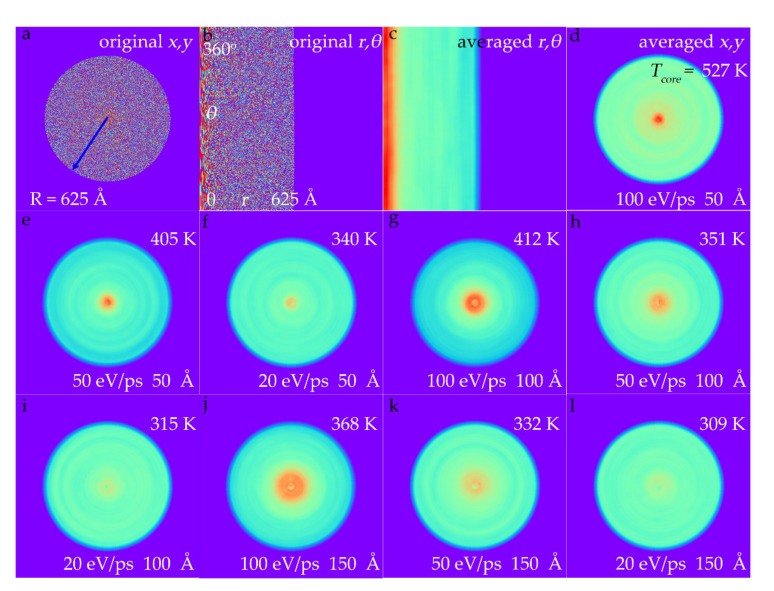
Thermal distributions of a circular graphene sheet exposed to the laser. (**a**,**b**) The equivalent temperature distribution of every atom in (**a**) Cartesian coordinates and (**b**) polar coordinates. (**c**) The averaged temperature distribution by a 10 × 30 kernel in polar coordinates. (**d**) The averaged temperature distribution in Cartesian coordinates transformed from (**c**). (**d**–**l**) Temperature distributions with different heat flow ranging from 20 eV/ps to 100 eV/ps, and different laser spot radii from 50 Å to 150 Å.

Vibrations of the circular graphene resonators are actuated by the laser spot. MD calculation is performed to study the actual temperature distribution of the graphene sheet with a radius of 625 Å when exposed to a laser, and the laser spot, with a radius of 50 Å, is opposite to the middle of the graphene. This middle area is a heat source, while the boundary of the graphene adheres to the zirconia ferrule substrate, serving as the heat drain. In this case, heat flows outward from the interior obeying Fourier’s law:

(6) $$\Delta Q=\kappa \frac{dT}{dr}2\pi rh$$

where Δ*Q* refers to the local heat flux; *κ* is the thermal conductivity; *dT*/*dr* is the temperature gradient; *r* is the radius to the center; *h* is the graphene sheet height, and 2π*rh* represents the cross-sectional area of heat flow. After the heat flow has reached a steady state, the velocities of every atom are recorded, the equivalent temperature of every atom is obtained using Equation (3), and the results are depicted in Figure 4a. Since the atom velocities are random and the temperature is the statistical outcome, a proper averaging strategy should be adopted to figure out the reasonable temperature distribution. Herein, the original temperature distribution (velocity distribution actually) is firstly transformed to polar coordinates as shown in Figure 4b. After that, a 10 × 70 kernel (about 5 thousand atoms in one kernel) is chosen to equalize the temperature distribution, and the outcome is shown in Figure 4c. It is clear that the temperature declines along the radius. Figure 4d shows the results reset to Cartesian coordinates. The temperature of the focus of the graphene sheet is much higher than other areas, peaking at 527 K, and the temperature drops steeply to 330 K at 150 Å. The high temperature existing in the 6% area in the middle is extremely likely to burn through the graphene sheet in practice. To avoid this, the heat flow should be controlled at an appropriate level. When the heat flow decreases to 50 eV/ps, the center temperature decreases to 405 K, and when the heat flow is 20 eV/ps, the center temperature drops to 340 K, which is quite a safe range. However, smaller heat flux means weaker actuation, which cannot be too low, in order to excite the mechanical vibration of the graphene resonator. To ensure enough actuation while avoiding overheating the center of the graphene, we were able to enlarge the laser spot radius instead. In practice, we could increase the distance between the end face of fiber and graphene to enlarge the area of the laser spot, or we could adopt a multimode fiber that has a bigger spot radius. Figure 4g–i show the temperature distribution with a heat source radius of 100 Å, and the focus temperatures decrease to 412 K, 351 K, and 315 K under heat flux values of 100 eV/ps, 50 eV/ps, and 20 eV/ps, respectively. When the heat source radius reaches 150 Å, the corresponding focus temperatures are 368 K, 332 K, and 309 K, which are in a reasonable range.

**Figure 5:**
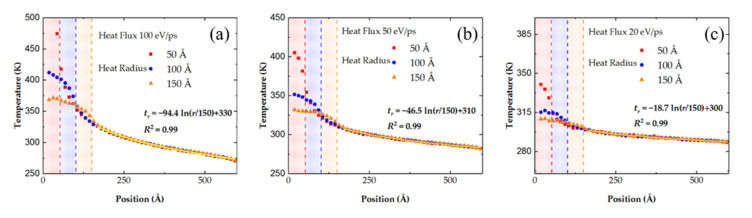
The temperature of graphene sheet at different positions from the interior to the boundary. The amount of heat flux is (**a**) 100 eV/ps, (**b**) 50 eV/ps, (**c**) 20 eV/ps.

To figure out the exact temperature distribution along the radius, the circular graphene sheet is divided into 50 chunks along the radius, and the temperatures of every chunk are shown in Figure 5. At the same heat flux, the graphene sheet with a larger heat area tends to have a lower maximum temperature, because the injected thermal energy is more scattered, thus avoiding being burned through. It is worth noting that the temperature curves outside the heating area with different heat radii but identical heat flux are overlapped, which is reasonable according to the heat flow equation. Based on Equation (6), the temperature outside the heating area can be expressed as

(7) $$T_{r}=T_{0}-\frac{\Delta Q}{2\pi \kappa h}\ln\left(\frac{r}{r_{0}}\right)$$

where *T_r_ T*_0_ are the temperatures at position *r* and *r*_0_, and other parameters are the same as Equation (6). Since the max heat radius is 150 Å, the temperature at this position is chosen as the reference temperature *T*_0_, and fitting curves match the MD calculation results quite well with an R-square of 0.99 as shown in Figure 5a–c. The scale factors are −94.4, −46.5, and −18.7, in direct proportion to the heat fluxes that are 100 eV/ps, 50 eV/ps and 20 eV/ps, respectively.

### 3.2. Mechanical Frequencies versus Adsorbates

**Figure 6:**
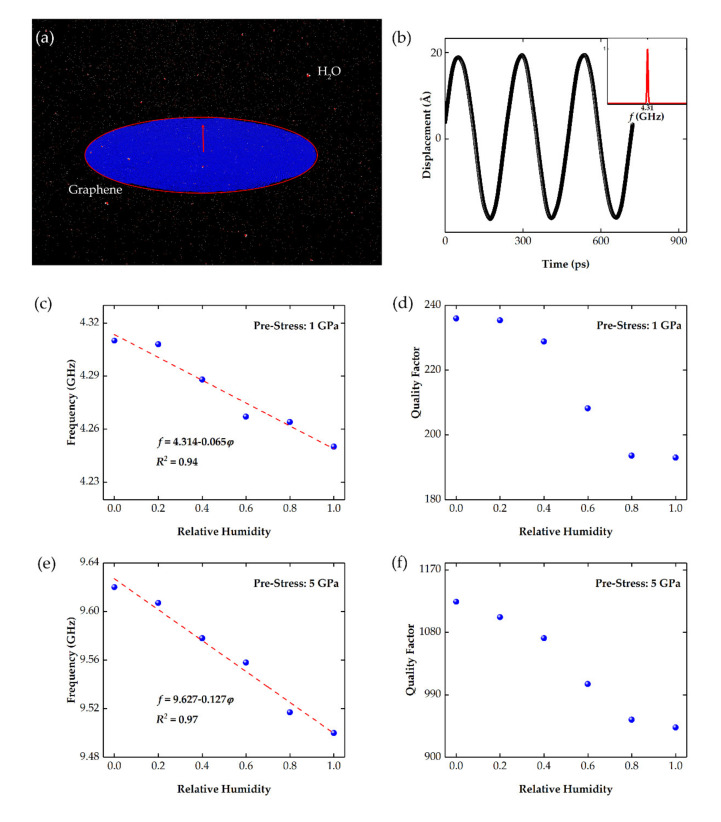
(**a**) The scheme for the MD simulation box. The diameter of the peripherally circular graphene sheet is 125 nm. The amount of water molecules around it is set to 0, 3.5, 6.9, 10.4, 13.8, 17.3 g/m^3^, corresponding to values of relative humidity of 0%, 20%, 40%, 60%, 80% 100% at 20 °C. (**b**) The displacement of the vibrating graphene sheet with pre-stress of 1 GPa, and the Fourier transform results show a frequency of 4.31 GHz. The mechanical frequencies (**c**,**e**) and quality factor (**d**,**f**) of graphene sheets with pre-stress of 1 GPa (**c**,**d**) and 5 GPa (**e**,**f**) under different environments with relative humidity values ranging from 0% to 100%.

After the thermally excited graphene sheet starts vibrating, the positions and velocities of every atom are calculated, and the corresponding mechanical frequencies are obtained by Fourier transform. Figure 6a presents the scheme of the MD simulation box with a size of 800 × 800 × 400 nm^3^. In the center, a peripherally circular graphene sheet with a diameter of 125 nm starts vibrating, and the average out-of-plane displacements are calculated, as shown in Figure 6b, and the Fourier transform is then performed to calculate the exact vibration frequency, as shown in the inset. It can be seen from Figure 6b that the maximum displacement of the graphene sheet is within 2 nm, which is far less than its size. Moreover, it should be noted that the tension in graphene would change a lot when transferred to the ferrule substrate, and this tension could also be regulated artificially by electrical and optical methods [9,12,44,45]. In the present MD calculation, the pre-stress in graphene is set to be 1 GPa and 5 GPa.

To investigate the effect of adsorbates on frequency shift, a mass of water molecules is put in the simulation box. The average spacing of water molecules can be deduced by the concentration of water vapor, and we can change the spacing of water molecules to represent different humidity. The relationship between average water molecule spacing and humidity at 20 °C is shown in detail in Table 1.

In this investigation, values of relative humidity of 0%, 20%, 40%, 60%, 80%, and 100% at 20 °C are considered. Figure 6c provides the MD calculated resonant frequencies of the graphene sheet with a tension of 1 GPa in different moisture. It is apparent from this picture that higher moisture lowers the resonant frequency, which is ascribed to the increase in the water molecules on the graphene resonator. According to the continuum elastics model, the mass-induced frequency shift follows

(8) $$\Delta f=-\frac{\Delta m}{2m_{0}}f_{0}$$

where Δ*m* is the adsorbate mass; *m*_0_ is the graphene mass, and *f*_0_ is the eigenfrequency of the graphene sheet, which is directly proportional to the square root of the tension of graphene, expressed as

(9) $$f_{0}=\frac{2.404}{2\pi r}\sqrt{\frac{\sigma }{\rho }}$$

where *r* is the radium; *σ* refers to the tension; and *ρ* is the density of graphene sheet. Based on this, a linear function is adopted to fit the MD dots, and the fitting results show that the slope is about 0.065, with an R-square of about 0.94. Since there are 468,552 carbon atoms in the suspended part of the graphene sheet, *m*_0_ in Equation (8) is about 9340 zeptograms. When the relative humidity rises from 0 to 100% at 20 °C, the mass of the total adsorbed water molecules is estimated to be 281 zeptograms. In this case, the mass sensitivity is calculated to be 214 kHz/zg. In consideration of the fact that the size of the MD model is one-thousandth of that of the real sensor, the actual sensitivity is estimated to be 214 Hz/zg, according to Equations (8) and (9).

On the other hand, the adsorbates would substantially degrade the quality factor of the graphene sheet, as shown in Figure 6d, as a result of the adsorption-induced material damping [46], and the interaction between the resonator and the water molecules also induces damping [20,47]. From Figure 6d, the quality factor of the graphene resonator is 236 without any adsorbates, and it decreases continuously with adsorption. When the relative humidity reaches 100%, the corresponding quality factor drops to 193; an 18% decline. To improve the quality factor, higher pre-stress is asserted to the graphene sheet, and the results are presented in Figure 6e,f. Higher stress leads to higher resonance frequency and mass sensitivity, reaching up to 487 kHz/zg, which is 2.3 times higher than that of the resonator with the stress of 1 GPa. More importantly, the quality factor reaches up to 1124 without adsorbates, an increase by nearly 4 times. At 100% relative humidity, the quality factor is still up to 943, much higher than the maximum quality factor of the resonator with the stress of 1 GPa. These results indicate that enlarging the pre-stress properly is an effective way to improve the mass sensitivity and the quality factor of the graphene resonator.

## 4. Conclusions

A scheme for an optical-thermally excited resonant mass detector is presented, and molecular dynamics calculations are performed to investigate the thermal actuation and resonant mass sensing mechanism. For the peripherally clamped circular graphene with a diameter of 125 nm (scaled to one-thousandth), MD results demonstrate that the maximum temperature of graphene exposed to the actuation laser is proportional to the laser power and inversely proportional to the laser spot radius. Typically, the temperature of the middle of the graphene reaches 527 K if the power of the actuation laser is 100 eV/ps and the spot radius is 50 Å. The high temperature occurs in the central 6% of the area, and in practice is extremely likely to burn through the graphene sheet. To avoid this, the power of the actuation laser should be controlled to within 20 eV/ps, or the actuation spot radius should be enlarged to 100 Å. In addition, when the graphene is actuated to vibrate, MD results show that the resonant frequency is 4.31 GHz for the graphene resonator with a pre-stress of 1 GPa, and the mass sensitivity is calculated to be 214 kHz/zg. Meanwhile, MD results reveal that the adsorption of water molecules degrades the quality factor quite a lot, from 236 to 193. However, stronger pre-stress could effectively improve both the mass sensitivity and the resonant quality factor, which increased by up to 1.3 and 4 times, respectively, for a graphene sheet with a pre-stress of 5 GPa.

## Figures and Tables

**Table 1:** The relation between average water molecule spacing and humidity at 20 °C.

Relative Humidity (%)	Absolute Humidity (g/m^3^)	Water Molecule Spacing (Å)
0	0	∞
20	3.5	204
40	6.9	163
60	10.4	142
80	13.8	129
100	17.3	120
